# Dislocation of the Humerus within the Axilla with Fracture of the Surgical Neck

**Published:** 1861-10

**Authors:** C. C. F. Gay


ART. III.—*Dislocation of the Humerus within the axilla with fracture of the surgical neck, by C. C. F. Gay.*

A maiden lady aged 63 was thrown from a sleigh January 26th, 1861, striking upon the left shoulder ; saw her half an hour after the accident. The arm was lying by the side of the body, pain and swelling at shoulder; numbness of fore-arm and hand; depression immediately beneath the accromian process; head of bone felt in axilla; crepitation. Placing the patient upon a settee in the recumbent position, a ball of cloth within the axilla and my heel upon the ball, made extension and counter-extension, assisted by Mr. Slocum, medical student, who manipulated at the shoulder. After using a moderate degree of force for about three minutes with a sweeping motion of the arm across the body, the head of the bone was felt to slip into its socket.

The position of the patient was such, the shoulder resting against the upright end of the settee, that the *counter-extending* was more than equal the *extending* force.

The patient now resuming the sitting posture, the arm was again ex amined and found to crepitate. The evidence was sufficient that crepitation did not arise from a fracture of the neck of the scapula, since the bone was in place and remained in place without any depression immediately beneath the accromian. I proceeded therefore to dress the arm as in ordinary case of fracture of the surgical neck, and in five weeks removed all dressings.

At the present time the arm is restored to its usual strength with impaired mobility, cannot raise the arm unassisted to a right angle with the body, nor place the hand in rear of the body.

*Remarks.*—I attributed the facility with which the reduction was effected to the extreme placidity of the muscles, the shortness of time which had elapsed and the assistance at manipulation rendered by Mr. Frink and Mr. Slocum. The bone I believe could have been reduced alone by manipulation, and I suspect the extending and counter-extending force would have been sufficient to have accomplished it.

Dr. Watson, of New York, reports a case reduced by the latter method—Dr. Hamilton accords greater efficacy to the former.

The older surgeons were in favor of first dressing the fracture and afterwards reducing the dislocation. The experience of the late surgeons, I believe, does not warrant this mode of procedure. Dr. Hamilton believes that the dislocation should first claim the attention of the surgeon, and that with the aid of anæsthetics its reduction in almost every case may be accomplished. Should an exceptional case occur, rather than leave it for after treatment, I would suggest that the plan proposed by Dr. Gray, viz: to cut down to the bone and replace it, admits of commendation and should be resorted to.

